# Patterning of Silicon Substrate with Self-Assembled Monolayers Using Vertically Aligned Carbon Nanotube Electron Sources

**DOI:** 10.3390/nano12244420

**Published:** 2022-12-11

**Authors:** Yi Yin Yu, Alfi Rodiansyah, Jaydip Sawant, Kyu Chang Park

**Affiliations:** Department of Information Display, Kyung Hee University, Dongdaemun-gu, Seoul 02447, Republic of Korea

**Keywords:** vertically aligned carbon nanotubes (VACNTs), electron beam lithography, patterning, self-assembled monolayer (SAM), octadecyl trichlorosilane (OTS)

## Abstract

We introduce a novel patterning technique based on e-beam lithography using vertically aligned carbon nanotube (VACNT) emitters with self-assembled monolayers (SAMs). A 20 μm line width of silicon wafer patterning was successfully demonstrated using octadecyl trichlorosilane (OTS) as a photoresist. To investigate surface modification by the irradiated electrons from the emitters, both contact angle measurement and energy dispersive X-ray (EDX) analysis were conducted. The patterning mechanism of the electron beam irradiated on OTS-coated substrate by our cold cathode electron beam (C-beam) was demonstrated by the analyzed results. The effect of current density and exposure time on the OTS patterning was studied and optimized for the Si wafer patterning in terms of the electronic properties of the VACNTs. The authors expect the new technique to contribute to the diverse applications to microelectromechanical (MEMS) technologies owing to the advantages of facile operation and precise dose control capability based on field electron emission current from the VACNT emitter arrays.

## 1. Introduction

Thanks to the development of sophisticated lithographic techniques, we are able to experience many advantages of electronic devices such as personal computers, laptops, cell phones, and electric vehicles. As the patterning techniques are elaborated, the memory capacity and performances of central processing units (CPUs) are drastically enhanced. For better performances of integrated circuits (ICs) and memory within a limited substrate area, a smaller feature size in the advanced lithography process is required [1,2].

There are various lithography tools available now such as conventional optical lithography [3,4,5,6], extreme ultraviolet (EUV) [7,8,9,10], electron-beam based lithography (EBL) [11,12,13,14], and X-ray lithography [15,16,17,18]. They have their own merits and demerits such as spatial resolution, throughput, and defects by mask contact; therefore, the use of the adequate tool is determined by the user’s considerations [1].

The lithography techniques can be classified by the types of light source and use of masks. Optical lithography is a conventional process using UV lights, and the wavelength of the UV source is reduced from 435.8 to 365.4 nm. Furthermore, a KrF laser source (193 nm) was introduced to reduce spatial resolution with a full field stepper [6]. The spatial resolution is determined by the wavelength of the light source, numerical aperture (NA) of the optic system, and Rayleigh constant [1].

To achieve less than 45 nm feature size of components in ICs, extreme ultraviolet lithography (EUVL) was devised [7]. Owing to the short wavelength of 13.5 nm and narrower FWHM characteristics, the technique is expected to be a key process for semiconductor manufacturing [19,20,21]. As of today, a feature size of 3 nm is capable using the process [22]. Due to the reduced wavelength, efforts to improve NA are critical for enhancing the spatial resolution. Commercialized EUVL systems have an NA of 0.33, which has been increased to 0.55 to improve production capacity, and the corresponding resolution is expected to be several nanometers [23].

The maskless lithography process is free from defects due to mask contact with the resist-coated substrate and byproducts from the mask itself. Electron beam lithography (EBL) is a maskless lithography process, the resolution of which is determined by resist due to the shorter electron wavelength (<0.1 nm). This direct writing of the focused electron beam on the substrates was able to not only realize 10 nm feature sizes [24] but able demonstrate complicated 3D nanostructures [25,26]. However, the light mass of electrons and accelerated voltage with magnetic focusing components result in low throughput and electromagnetic noise. To cope with the restricted exposure area of the single-electron beam source of EBL, a matrix of multiple electron beam sources is needed. Cold cathode-based electron emitters are applicable to multi-electron sources by fabricating numerous emitters on a large area substrate, and an M × N matrix type emitter source can be prepared by modularization of the diode or triode configuration of emitter sources. Moreover, one of main drawbacks of the EBL process is scattered electrons within the resist [27]. The scattering electrons result from deterioration of the spatial resolution by smearing to unwanted positions in the resist. Therefore, heavy ions can be substituted to overcome this phenomenon in so-called ion-beam lithography [28,29,30,31]. In the current EBL technique, a single focused electron beam is exposed on a limited area of substrate; thus, stitching of the beam by raster or scanning is required. Extensive time is needed for covering the whole substrate. However, cold cathode M × N matrix-type multi-electron emitters can extensively reduce the process time by fabricating scaled up emitter arrays. Furthermore, matrix-type cold cathode modules can cope with the large area substrate.

In terms of resist for EBL, the thickness is critical to achieve the desired feature size due to the smearing artefact by electron beams. To overcome this drawback, self-assembled monolayers (SAMs) have been intensively studied. The application of representative SAMs for EBL has been reported elsewhere in detail [32]. The drastically reduced thickness of the SAMs enables precise pattern definition by eliminating the artefact while enhancing sensitivity to the irradiated electrons. Furthermore, the thin layers in the application for SAMs have special properties that can be tailored such as adhesion resist for specific attachment at the molecular level.

**Figure 1:**
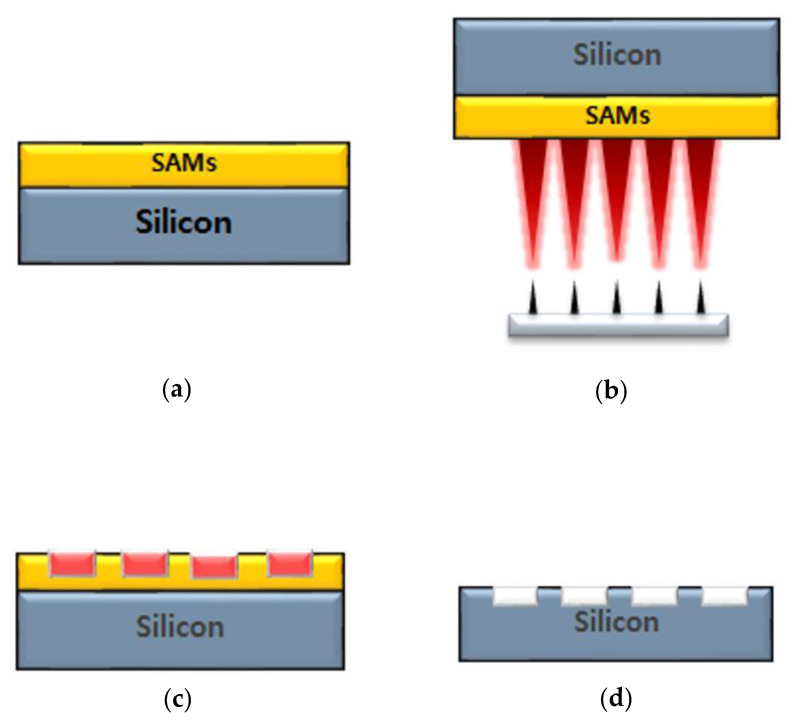
Sequential procedure of Si substrate patterning by vertically aligned carbon nanotube electron sources: (**a**) coating of OTS on silicon substrate; (**b**) C-beam irradiation on OTS-coated Si substrate by vertically aligned carbon nanotube (VACNT) electron sources in diode configuration; (**c**) structural modification of OTS after C-beam exposure; (**d**) etched Si wafer after C-beam exposure with KOH solution.

In this report, we focus on the EBL process by using a combination of vertically aligned carbon nanotubes (VACNTs) as the electron source and octadecyl trichlorosilane (OTS) as SAMs without any electromagnetic focusing components. Due to the direct writing of electrons on the resist, the maskless process, and the capability of selective growth of the VACNTs at the desired position, 20 μm line patterns can be easily achieved. The Si patterning process of OTS coated substrate by VACNT emitters is represented in Figure 1a–d. For simple driving of the electron emitters, a diode configuration is used for practical electron beam operation. With a 200 × 200 μm single square emitter array, 20 μm line patterning of Si substrate is demonstrated. A resist OTS is used, and, to reveal the patterning mechanism of OTS by irradiated electrons, contact angle measurement and compositional analysis are conducted. The authors anticipate that this facile but sophisticated patterning technique will be conducive to next-generation e-beam-based lithography processes for MEMS and nano-electric device applications.

## 2. Materials and Methods

### 2.1. SAM Sample Preparation

The SAM-coated Si substrates were prepared by dipping the wafer in octadecyl trichlorosilane (OTS) solution. The OTS was purchased from Sigma Aldrich Chemical Co, Inc. (90% purity) (St. Louis, MO, USA). The SAMs were coated by immersing the silicon wafer in OTS/toluene 1:200 *v*/*v* solution for 4 h. To avoid contamination, the silicon wafer was prepared by pre-soaking in acetone and isopropyl alcohol in an ultrasonic bath for 10 min each. After immersing the substrate in OTS/toluene solution, the samples were rinsed with pure toluene solvent and blown with nitrogen. The detailed preparation procedures were described elsewhere [31].

### 2.2. Fabrication of VACNT Emitter

**Figure 2:**
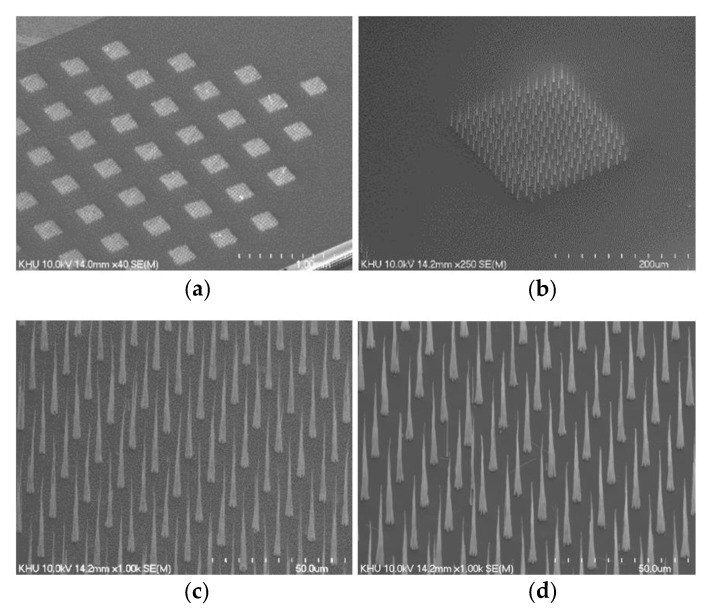
SEM images of the two groups of emitter arrays: (**a**) partial image of 88-emitter array and (**b**) single-emitter array; (**c**,**d**) represent magnified SEM images of (**a**,**b**) at the same magnification of 1000. The side length of the squares in (**a**,**b**) is 200 μm.

Vertically aligned carbon nanotube (VACNT) emitters were grown on n-type silicon substrate [33]. We used 30 nm thick Ni as the catalyst layer, which was deposited onto the silicon substrate by sputtering. In turn, the Ni-etched Si wafers were loaded unto our homemade triode-type direct current plasma-enhanced chemical vapor deposition (dc-PECVD) chamber for VACNT growth. C_2_H_2_ and NH_3_ gases were fed into the chamber at elevated temperature. To dissociate the feeding gas and promote CNT growth, the plasma was ignited by biasing voltage at the cathode and the gate electrode at −600 and 300 V, respectively. Detailed growth conditions are described in Table 1. The process was optimized by considering the area of the Ni catalyst. We prepared two groups of emitter arrays for the analysis of electrical properties, surface modification evaluation, and practical Si substrate patterning. The first group had an 88-island emitter array with a square shape, referred to as group A, as shown in Figure 2a. One island consisted of 14 × 14 emitter tips with 15 μm pitch, and the length of the square was about 200 μm. Then, a single-island emitter array was fabricated, named group B, for Si substrate patterning. An SEM image of the group B emitter island is given in Figure 2b. The numbers and positions of islands could be controlled by the Ni etching process.

The VACNT emitters were fabricated as a cone shape with an average height of 42 μm and dot diameter of 3 μm at the bottom of the emitters. After growth, the as-grown CNTs were post-treated with HF to enhance emission [33]. To evaluate the electrical properties of the emitters, we conducted I–V measurements with a diode configuration using a Matsusada power supply for anode biasing at 5 kV. The current was measured using a multimeter, Agilent 34401A, and the measurement was conducted in vacuum conditions under 2 × 10^−7^ Torr.

### 2.3. OTS Patterning and Si Wafer Patterning

**Figure 3:**
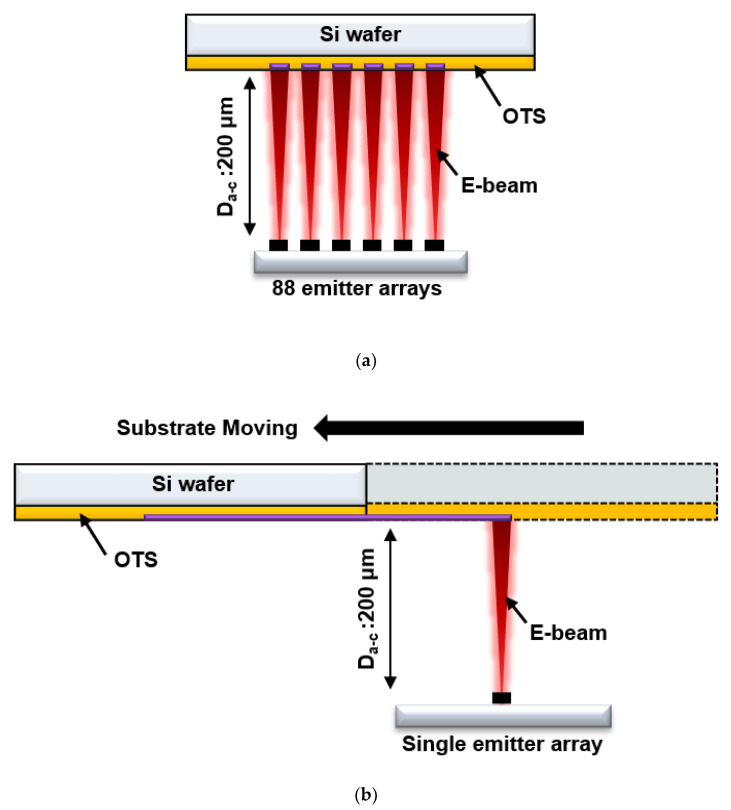
Schematic diagrams for (**a**) water contact angle measurement and EDX mapping analysis using an 88-island emitter array of VACNTs and (**b**) line patterning of Si wafer using VACNT single-island emitter array.

To understand the structural and compositional modifications of the OTS by irradiated electron beams, an 88-island emitter array of VACNTs was used. The OTS-coated substrate and emitters were loaded inside our high-vacuum chamber for electron irradiation. The schematic of diode configuration is depicted in Figure 3a. The gap between emitter tip and OTS surface was maintained at 200 μm. Anode bias was applied by Matsusada, and the current was measured using an Agilent 34401A digital multimeter. The exposure time was varied from 10 to 40 s to determine the optimized condition of OTS pattern definition, and the current density effects were also investigated. By varying the exposure times of electrons and current densities, the patterning mechanism of OTS layer was explored. Water contact angle measurement and EDX mapping were conducted to investigate the structural changes of the OTS. Additionally, for practical Si wafer patterning, a single-island emitter array of VACNTs was used. To get the line pattern of OTS, the OTS-coated Si substrate was moved in a lateral direction at 0.06 mm/min, while the VACNT emitters was fixed. The scheme of electron irradiation for Si patterning is described in Figure 3b. After formation of the OTS patterning, the substrate was immersed in KOH 45% (*v*/*v*) solution at 70 °C for 1 h for Si wafer etching.

## 3. Results and Discussion

**Figure 4:**
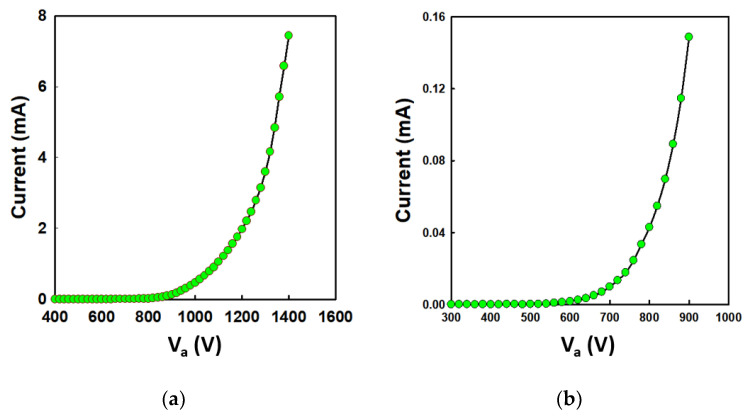
I–V curves of (**a**) 88-island emitter array and (**b**) single-island emitter array in a diode configuration.

Prior to exposing electrons on OTS-coated substrates, the I–V characteristics of emitter arrays were evaluated. Figure 4a shows the I–V characteristics for the group A emitter array. The maximum current achieved was 7.4 mA at 1.4 kV of anode voltage with 700 V as the threshold voltage. The I–V curve for the group B emitter is shown in Figure 4b, with a maximum current of 0.14 mA at 900 V. The turn-on voltage for the group B emitter was 580 V.

**Figure 5:**
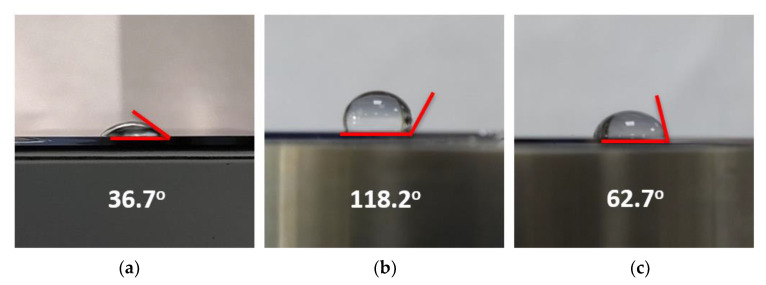
Water contact angle measurement for (**a**) bare silicon substrate, (**b**) silicon substrate deposited by OTS, and (**c**) silicon substrate after C-beam irradiation using 88-island emitter array of VACNTs.

**Figure 6:**
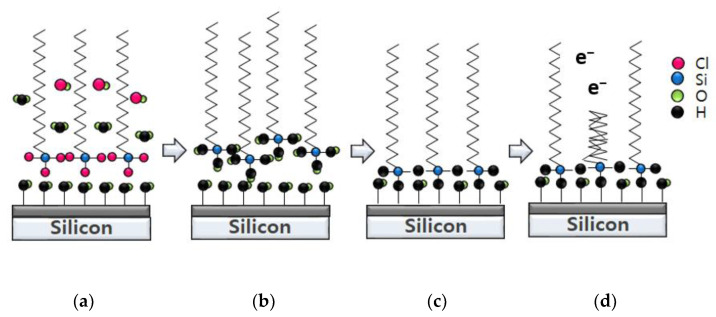
Mechanism of OTS patterning by C-beam: (**a**) OTS physisorption on Si substrate after coating; (**b**) HCl desorption by Si substrate surface and OTS reaction; (**c**) Si−H bonding and hydrophobic terminal formation; (**d**) bond breaking by electron beam and weakened hydrophbic property.

To analyze the structural modifications of the SAM surface after electron beam irradiation, we carried out water contact angle measurements. For the OTS surface modification, we irradiated the electron beam with an emission current of 1 mA and anode bias of 1.1 kV at the same exposure time for 20 s using the 88-island emitter array. Figure 5a–c show the comparison of water contact angle for the bare silicon substrate, silicon substrate deposited by OTS, and silicon substrate after exposure to the C-beam. The water contact angle of silicon wafers after SAMS coating was 118.2°. On the other hand, the water contact angle of bare silicon was 36.7°. The higher water contact angle of silicon after OTS coating indicates that the silicon interface had modified surface properties after OTS deposition. The OTS layer made the surface of silicon more hydrophobic. Thus, the water contact angle after OTS deposition was larger than that of bare silicon substrate. After exposure to the C-beam (Figure 5c), the water contact angle was reduced to 62.7°. Kanan reported that the reduction in water contact angle was related to distinct changes in surface energy at the interfaces [34]. The water contact angle decreased due to the electron irradiation by breaking the bonding structure on the OTS layer. The ambient interface might be changed from layers of OTS containing –CH_3_ groups to those containing –CH_2_ groups. This may have also been due to a change in most OTS terminal groups from hydrophobic (containing –CH_3_) to hydrophilic (containing polar groups, e.g., –OH and –COOH). A brief illustration of the mechanism of electron beam interaction with the OTS substrate is shown in Figure 6.

Initially, the OTS was layered onto the silicon substrate via a physisorption mechanism (Figure 6a). During this reaction, the Cl from OTS reacted with the H from silicon substrate to become HCl. As a result, the water was absorbed, thereby reducing the contact angle, i.e., hydrolysis. Furthermore, OTS could be perfectly organized and assembled by covalent grifting, as shown in Figure 6b. The head site of SAMs attached to the substrate, leaving the tail site behind. As a result, the hydrophobic properties developed, as shown in Figure 6c. When the electrons bombarded onto OTS, the chain has cut. Hence, the electron beam-irradiated region led to an opening on the OTS structure. This change in structure is shown in Figure 6d, resulting in the OTS no longer being hydrophobic. As a result, a reduction in water contact angle after C-beam exposure was observed.

**Figure 7:**
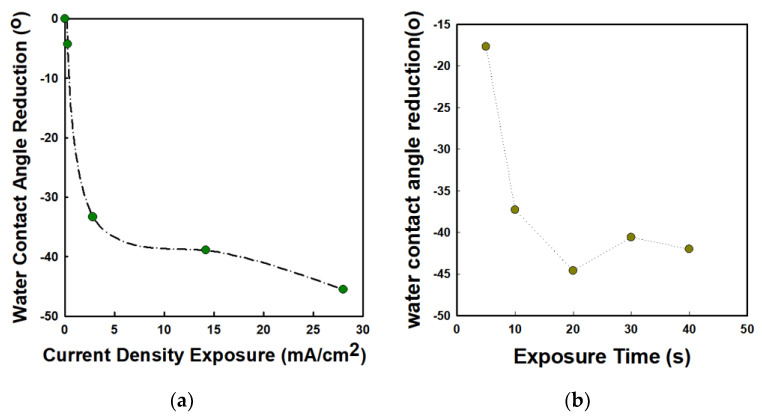
Water contact angle measurement with respect to (**a**) current density and (**b**) exposure time. The exposure time and current were fixed at 20 s and 0.28 mA/cm^2^, respectively.

**Figure 8:**
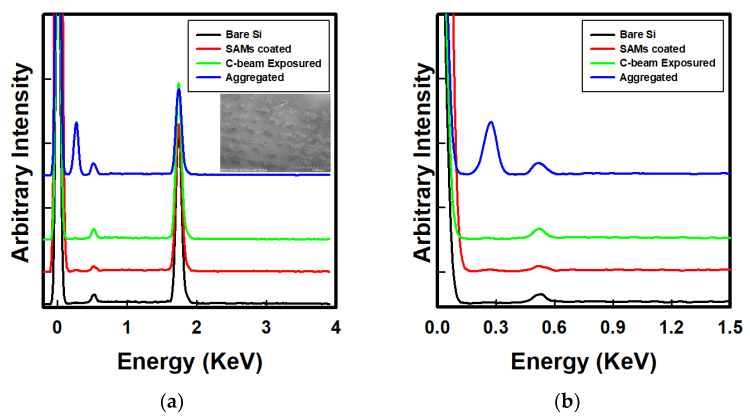
(**a**) EDX spectra of substrates after electron beam irradiation with 88-island emitter array on the designated positions, where aggregated carbon islands are visible in the inset. (**b**) Enlarged plot under 1.5 kV acceleration voltage.

Through water contact angle measurement, we investigated the surface modification under various exposure conditions. We varied the current density from 0.28 mA/cm^2^ to 28 mA/cm^2^ at a fixed exposure time of 20 s. The current density of the 88-emitter array was calculated by dividing the measured anode current by the total emitter area (0.002 × 0.002 × 88 cm^2^). As shown in Figure 7, the water contact angle was reduced significantly by increasing the current density of exposure. With an increase in the current exposed to the OTS-coated substrate, the water contact angle decreased. At the current density of 28 mA/cm^2^, the largest reduction in water contact angle by 46.5° was confirmed. For the 2.8 mA/cm^2^ current density exposure, the reduction angle was about 38.9°. For the exposure to the lowest current density of 0.28 mA/cm^2^, the reduction in water contact angle was only 4.3°. In addition to the investigation of compositional changes and surface modification of OTS after C-beam exposure, scanning electron microscopy (SEM) and energy-dispersive X-ray analysis (EDAX) were conducted. The inset in Figure 8 shows the SEM image of OTS after C-beam exposure to 28 mA/cm^2^ using the 88-island emitter array of VACNTs. As shown in Figure 8, a distinct dark area of the SAM surface after exposure to the C-beam can be observed. As reported by Kanan [34], the burned area represents surface contamination due to enhanced adsorption at the OTS–substrate interface. Therefore, the electrons from the C-beam could increase the surface energy in the exposed region on the SAMs. The dark region was similar in shape to the emitter island, confirming that electrons were only emitted from the CNT area. The EDX mapping analysis results suggest that the dark spots may have resulted from differential charging of the surface or from differential scattering of secondary electrons [34]. Furthermore, we performed an EDAX mapping measurement and confirmed the elemental contents in the OTS sample before and after exposure to the C-beam. Figure 8a,b show the EDAX spectrum of SAMs before and after electron beam irradiation by the 88-island emitter array. In the energy range from 0.28 to 0.57 keV, carbon-related peaks were confirmed, except for the bare Si wafer. A remarkably increased carbon peak was distinguishable at 0.28 keV, which was attributed to aggregated carbon in the overexposed region of OTS. In addition, a slight increase in the broad band was observed for the OTS-coated Si wafer. However, the intensity was reduced slightly after electron beam irradiation. The remaining carbon peak was ascribed to inherent carbon chains within the OTS layer. Specific information on EDX analysis results is presented in Table 2.

As shown in Table 2, there was no carbon on the bare silicon substrate before deposition of OTS. After OTS coating, the carbon peaks appeared, highlighting the presence of this element in OTS. The amount of carbon after OTS deposition was about 13.24%. The total amount of carbon might have stemmed from the alkyl chain of OTS. Furthermore, we also confirmed the EDAX spectrum for the substrate after exposure at a current density of 28 mA/cm^2^. In that case, significant peaks appeared in different regions. The region of exposure had a reduction in carbon amount. The intensity of the carbon peak was no longer higher than that before exposure. The total amount of carbon after exposure became 7.11%. However, we noticed a bulk amount of carbon on some sites after 28 mA/cm^2^ current density exposure. We expect that these sites might have been due to the overexposed area which featured some carbon aggregation and left an empty site after shrinking, with these sites accounting for 58.32% of the carbon detected. To fabricate the micro-patterning of silicon substrate, we used the C-beam from one island shape (Group B) as the electron source. For the pattering, we used 0.28 mA/cm^2^ of exposure current density and varied the duration of e-beam exposure from 5 to 40 s. The relevant water contact angle measurement results are given in Table 3. As shown in Table 3, a longer exposure time resulted in more damage to the SAM layer. The optimum exposure time was 20 s, with the highest reduction in water contact angle (44.6°) compared with 30 s (40.6°) and 40 s (42.0°), although this difference was not significant. We considered 20 s as the optimum exposure time for breaking bonds in OTS while minimizing damage or aggregation of the carbon network within OTS.

**Figure 9:**
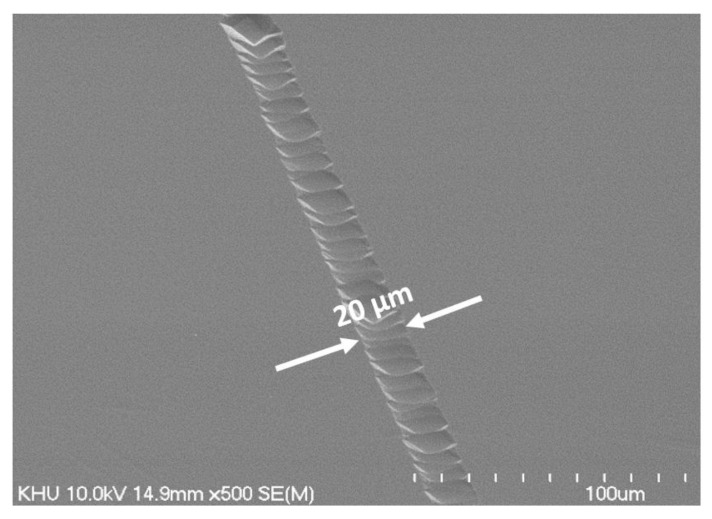
SEM image of Si wafer etched with KOH solution after OTS patterning using a single-island emitter array with 14 × 14 emitter tips.

**Figure 10:**
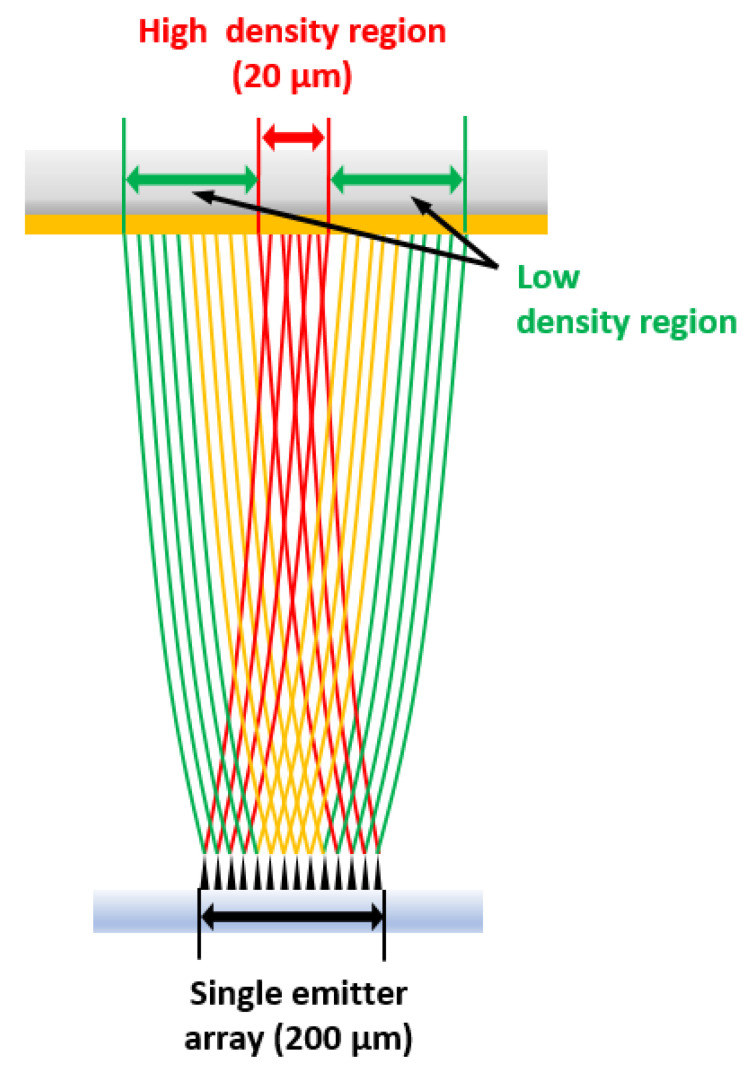
Electron beam trajectories using diode configuration of VACNTs and classification of dose regions for effective patterning of OTS.

Figure 9 shows the etched silicon substrate after electron beam irradiation by the single-island emitter array of VACNTs on OTS. Sequential electron beams were exposed on the OTS-coated Si wafer at 0.28 mA/cm^2^ of current density for 20 s while manually moving the substrate horizontally at 0.6 mm/min. Due to the overlapping of electron beam shots, superimposed joints can be seen within the line groove. The width of the etched groove was about 20 μm according to SEM observation. The dimension of the etched Si width was one-tenth of the single-island emitter array’s length (200 μm). The reduced dimension of the Si substrate compared to the emitter is depicted in Figure 10. The C-beam-exposed area could be divided into two regions. At the center of the emitter island, electron emission current was superimposed by the anode bias of 1 kV with sufficient density for OTS patterning but peripheral areas had insufficient current density to break bonds in OTS. Therefore, only the narrow area of OTS was developed and exposed to KOH etchant, resulting in Si patterning.

## 4. Conclusions

We successfully demonstrated Si wafer patterning using vertically aligned carbon nanotube (VACNT) electron emitters with OTS as the resist. Water contact angle measurements proved that structural changes occurred in the OTS layer by breaking carbon bonds upon electron beam irradiation. Proper dose conditions for OTS patterning were explored by varying the electron current density and exposure time of C-beams. Redundant exposure of the electron beam resulted in aggregation of the carbon network, and the mechanism was revealed by EDX mapping after exposure. At the optimized current density of 28 mA/cm^2^ and exposure time of 20 s, Si patterning was successfully demonstrated. Considering the emitter dimension of 200 μm and the patterned width of 20 μm on the Si substrate, one-tenth of the patterning feature was achieved. The center of the C-beam had higher electron density than the peripheral region, resulting in focused OTS patterning.

In summary, we could achieve 20 μm of Si wafer patterning using a combination of 200 μm wide VACNT electron emitters and OTS as a resist without any focusing elements; we believe that this technique will be helpful for advanced EBL technologies.

## Figures and Tables

**Table 1:** Growth condition of VACNT emitters.

VACNT Emitter Growth Condition							
Group	Description	Temp. (°C)	Pressure (Torr)	Time (min.)	C_2_H_2_:NH_3_ (sccm)	Gate Voltage (V)	Cathode Voltage (V)
A	88-island array	850	1.8	90	16:140	300	−600
B	Single-island array	850	2.2	90	16:140	300	−600

**Table 2:** Summary of EDAX atomic analysis of various substrates with electron beam exposure.

Element	Area			
	Si Substrate	OTS As Deposited	After C-Beam Exposure	Aggregated Area
Carbon (C)	0	13.24	7.11	58.32
Oxygen (O)	9.32	10.18	11.41	11.7
Silicon (Si)	90.68	76.58	81.48	29.91

**Table 3:** Water contact angle as a function of exposure time at 0.28 mA/cm^2^ current density.

Exposure Time (s)	Contact Angle (°)	
	Before	After
5	93.6	75.9
10	102.6	65.3
20	103.9	59.3
30	104.9	62.9
40	104.9	62.9

## Data Availability

The data presented in this study are available on request from the corresponding author.
